# Spasmodic Dysmenorrhœa

**Published:** 1902-05-24

**Authors:** 


### SPASMODIC DYSMENORRHŒA.

The matter is otherwise, however, in regard to spasmodic dysmenorrhœa. Menstrual pain of extreme severity is almost always of the spasmodic kind. It is common for patients to say that when it is present they cannot lie still, they roll about with pain, it doubles them up, keeps them awake at night, and makes them perspire and sometimes faint. In about one-fourth of the cases it causes vomiting. Its usual duration is less than 24 hours, and it only exceptionally lasts longer than two days. But patients who suffer from uterine congestion may have two kinds of pain, the aching due to pelvic congestion which may precede the flow by several days and the spasms of uterine colic which only last a few hours, and this is a matter which must be inquired into, as women may not always properly distinguish between the two. The pain of spasmodic dysmenorrhœa comes in sudden, sharp pangs, and between these twinges there is either no pain or a dull general aching in the pelvic region. Each attack of pain is of short duration, usually only a minute or two. The natural cure of spasmodic dysmenorrhœa is by pregnancy. The best drugs for the relief of uterine colic, says Dr. Herman, are antipyrin and phenacetin. In slight cases they give adequate relief, and as we know not of any harmful results from their occasional use, if these drugs relieve there is no need for further treatment. If these drugs fail, powerful narcotics may be required, but it is a bad thing for a young woman to use these drugs every month, and if this becomes necessary local treatment is preferable. The local treatment of spasmodic dysmenorrhœa is to dilate the cervix by the passage of bougies. In a few cases 10 grains of guaiacum resin in a tablespoonful of malt extract, twice or three times a day, beginning a week before menstruation is expected, will remove the pain and sometimes will cure. How it acts, and in what cases it will succeed are unknown.

